# Comparison of SPECT/CT, AEEG, and 3D-pCASL in people with epilepsy

**DOI:** 10.3389/fneur.2025.1633993

**Published:** 2025-08-20

**Authors:** Rong Chen, Qing Zhang, Yajun Li, Jingyun Chen, Xu Wang, Yanzi Jin, Fang Li, Qiuyan Chen, Mengyun Li

**Affiliations:** ^1^Department of Neuroelectrophysiology, Cardiovascular and Cerebrovascular Disease Hospital Branch, General Hospital of Ningxia Medical University, Yinchuan, Ningxia, China; ^2^Department of Neurology, General Hospital of Ningxia Medical University, Yinchuan, Ningxia, China; ^3^Clinical Medical College, Ningxia Medical University, Yinchuan, Ningxia, China

**Keywords:** PWE, SPECT/CT, AEEG, 3D-pCASL, positive detection rate

## Abstract

**Objective:**

To analyze the clinical value of single-photon emission computed tomography (SPECT)/computed tomography (CT), ambulatory electroencephalogram (AEEG) and three-dimensional pseudo-continuous arterial spin labeling (3D-pCASL) in people with epilepsy (PWE).

**Methods:**

The study included 98 PWE who were treated in The General Hospital of Ningxia Medical University from December 2021 to September 2023. The positive detection rates and characteristics of SPECT/CT, AEEG and 3D-pCASL were compared. Additionally, the agreement between SPECT/CT, 3D-pCASL and AEEG-positive results was analyzed.

**Results:**

The positive detection rates for SPECT/CT, AEEG, and 3D-pCASL examinations were 77.55% (76/98), 62.24% (61/98), and 54.08% (53/98) respectively. SPECT/CT exhibited higher positive detection rates than AEEG (aOR = 1.165, 95%CI: 1.036–1.311, *p* = 0.011) and 3D-pCASL (aOR = 1.265, 95%CI: 1.119–1.430, *p* < 0.001), no significant difference was observed between AEEG and 3D-pCASL (aOR = 0.922, 95%CI: 0.810–1.048, *p* = 0.213). In combined modalities, the positive detection rate of AEEG+SPECT/CT + 3D-pCASL (93.88%, 92/98) was higher than AEEG+3D-pCASL (79.59%, 78/98, *p* = 0.003), but showed no statistically significant difference compared to AEEG+SPECT/CT (88.78%, 87/98, *p* = 0.204). The proportions of PWE with no lesions, focal lesions, unilateral multifocal lesions and bilateral lesions across the three modalities results revealed statistically significant differences (*p* < 0.05). Among 61 PWE with interictal epileptiform discharges (IEDs) detected by AEEG, the agreement rate between SPECT/CT and AEEG was 63.93% (39/61), while that of 3D-pCASL was 39.34%(24/61), the difference is statistically significant (*p* = 0.007). In PWE with AEEG-detected temporal lobe (43 cases) and frontal lobe (31 cases) IEDs, SPECT/CT exhibited higher agreement rate with AEEG compared to 3D-pCASL (*p* < 0.05).

**Conclusion:**

SPECT/CT demonstrated a high positive detection rate and showed strong agreement with AEEG-positive results in PWE. The combined use of SPECT/CT, AEEG and 3D-pCASL significantly increased the positive detection rate. These findings highlight the advantages of SPECT/CT and combined modalities.

## 1 Introduction

Epilepsy is a chronic condition and one of the most prevalent neurological disorders, characterized by recurrent abnormal neuronal discharges that lead to temporary dysfunction of the central nervous system (CNS). Seizures severely impaired the quality of life of both PWE and their families (1). Currently, electroencephalogram (EEG) has been widely utilized in epilepsy diagnosis due to its simplicity, cost-effectiveness, and high sensitivity (2). Since the 1970s, AEEG has been frequently used. This portable recording system extends monitoring time compared to EEG, offering distinct advantages in epilepsy diagnosis and lesion localization (3).

Epilepsy is often accompanied by a series of physiological and biochemical alterations, including hemodynamic changes and cerebral metabolism abnormalities. Consequently, functional neuroimaging techniques play a unique role in epilepsy. Common functional neuroimaging techniques include positron emission computed tomography (PET), SPECT, magnetic resonance spectroscopy (MRS), blood oxygen level-dependent functional magnetic resonance imaging (BOLD-fMRI), arterial spin labeling (ASL), and CT perfusion imaging (CTP). SPECT is a nuclear medicine modality that reflects the functional status of organs or tissues. SPECT/CT integrates functional SPECT with anatomical CT imaging, combining metabolic and structural information to facilitate precise anatomical localization of functionally abnormal regions. The localization value of SPECT/CT in PWE is based on the coupling of cerebral metabolism and perfusion (4). By using imaging agents to visualize regional cerebral blood flow (rCBF), distinct pathophysiological changes in rCBF and metabolism during ictal and interictal phases can be observed: the epileptic lesion areas exhibit reduced cortical perfusion during interictal periods and increased perfusion during seizures (5). ASL is a powerful noncontrast MRI technique for evaluation of rCBF. Three predominant ASL methodologies currently employed in neuroimaging include Pulsed Arterial Spin Labeling (PASL), Pseudo-Continuous Arterial Spin Labeling (pCASL), and Velocity-Selective Arterial Spin Labeling (VSASL). Compared to PASL, PCASL demonstrates distinct advantages by permitting extended arterial labeling durations and ensuring proximal spin labeling to the tissue of interest, thereby theoretically enhancing the signal-to-noise ratio (SNR) of ASL sequences by a factor of √2. The principal advantage of VSASL compared to the other two techniques lies in its capacity for ultra-proximal spin labeling, which substantially reduces arterial transit time (ATT) (6). Advanced neuroimaging modalities often improve epilepsy surgical outcomes by contributing key information during the highly individualized surgical planning process and intraoperative localization, including SPECT/CT and ASL (7). The post-labeling delay (PLD), defined as the time interval between arterial water labeling and cerebral blood flow measurement, represents a critical parameter in ASL. Multi-delay ASL (MDASL) utilizes multiple PLDs to enhance the accuracy of arterial transit time (ATT)-corrected rCBF quantification. PLD selection depends on estimation of the mean ATT from the labeling plane to tissue compartments. Optimal PLD is established within an ideal temporal window immediately following the maximum ATT in a given patient, thereby maximizing signal detection from all labeled protons (8). Ictal and interictal SPECT scans can be comparatively analyzed inPWE. Ictal phase images are processed using subtraction algorithms that remove activity from interictal (i.e., background) scans, generating subtraction (ictal minus interictal) SPECT. This subtraction technique improves localization of the seizure focus (7). Integrated PET/MRI protocols enable precise assessment of cerebral anatomy and structural abnormalities while concurrently acquiring metabolic information during a single imaging session. This approach significantly reduces radiation exposure, sedation duration, and procedural sedation requirements. Brain PET/MRI has demonstrated particular utility for accurate localization of epileptogenic zones in pediatric epilepsy cases, providing critical supplementary data that guides surgical planning for medically refractory patients (9). The application of deep learning in epilepsy neuroimaging has demonstrated significant utility, particularly for preoperative evaluation of medically refractory epilepsy (10). With rapid advancements in SPECT and ASL neuroimaging for epilepsy management, a comparative analysis was conducted of SPECT/CT, AEEG, and3D-pCASL findings in 98 PWE to evaluate their clinical utility.

## 2 Methods

### 2.1 Subjects

The study subjects were 98 PWE treated at The General Hospital of Ningxia Medical University from December 2021 to September 2023. General patient data, AEEG results, interictal SPECT/CT and 3D-pCASL imaging results were collected. In addition to meeting the diagnostic criteria for epilepsy established by the International League Against Epilepsy (ILAE) in 2014, inclusion criteria for participants included: (1) No contraindications to SPECT/CT or MRI; (2) Absence of structural brain abnormalities such as traumatic brain injury, space-occupying lesions, or encephalomalacia; (3) Voluntary agreement of the subjects or their guardians to participate in the study, along with the ability to provide comprehensive case data.

The study was approved by the Local Ethics Committee of the General Hospital of Ningxia Medical University (Approval No. KYLL-2021-1087) and obtained informed consent from all study participants.

### 2.2 SPECT/CT date

Interictal SPECT/CT acquisitions were conducted ≥24 h after the last seizure. Scans were performed using dual-probe SPECT/CT instrument (Discovery NM/CT 670 Pro GE, USA). 99mTc-ethyl-cysteinate dimmer (99mTc-ECD) was prepared from a commercial kit (Wuxi Jiangyuan Industry Technology and Trade Co, China) and was marked and quality controlled by The Department of Nuclear Medicine at the General Hospital of Ningxia Medical University, with a radiochemical purity greater than 98%. Scans were performed within 30 min of tracer preparation. Imaging commenced ≥30 min post-injection in a dimmed and quiet environment. The dose was calculated according to the age {[(age+1)/(age+7)] × 370 MBq}. 128 images of 25 s duration in a 128 × 128 matrix were obtained over 360°. The total acquisition time was 28 min. Subsequently, a routine scan of the head CT was performed, with a matrix of 256 × 256, a magnification of 1, and a slice thickness of 5 mm.

With a cut-off frequency of 0.35–0.40 cycles/cm, and the original images were reconstructed into cross-sectional images, coronal images, and sagittal images. Two nuclear medicine physicians independently interpreted the SPECT/CT studies. Utilizing cross-sectional images as a primary reference, and considering information from the coronal and sagittal cross-sections, with bilateral comparisons, the identification of two consecutive levels displaying either decreased or increased perfusion was considered indicative of positive foci. Image interpretation was performed without knowledge of AEEG results.

### 2.3 AEEG date

AEEG monitoring was performed with electrodes positioned according to the International 10–20 System, with bipolar montages monitored (bilateral fronto-anterior temporal, anterior temporal-central, central-parietal, and parietal-occipital). Monitoring typically lasted 24 h, capturing static, dynamic, and sleep-stage-specific EEG activities. A 20-min resting-state recording (eyes-closed) served as baseline reference. All subjects underwent the same standardized elicitation procedures (Eye Opening/Closure, hyperventilation). Two independent clinical neurophysiologists interpreted the recorded results for each case. AEEG positivity required consensus-confirmed IEDs (spikes, sharp waves, or spike–wave complexes).

Eye Opening/Closure: Should be performed per standard protocol: patient awake and relaxed, starting with baseline recording eyes closed (typically 1 min), followed by eyes open fixation on a target (10–15 s), then eyes closed recovery (30 s–1 min), repeated several times. Precise EEG marking at each state transition is critical.

Hyperventilation (HV): Should be performed per standard protocol: Strict screening for contraindications, patient in a sitting position, performing deep and rapid breathing (target rate 20–25 breaths/min), for a duration of 3 min (shorter for children or frail patients), followed by a 3–5 min eyes-closed recovery period. If the HV provocation test is deemed suboptimal, it could be repeated once after an interval of at least 5 min. Precise marking at the start and stop of HV, and the start/end of recovery is equally essential.

### 2.4 3D-pCASL date

Interictal 3D-pCASL acquisitions were conducted ≥24 h after the last seizure. All scans were performed using a 3.0 T MRI scanner (GE Signa Architect) with a 48-channel phased-array head coil. Participants were positioned supine with eyes closed, maintaining quiet respiration and minimizing movement. The imaging protocol commenced with structural sequences - including axial spin-echo T1-weighted imaging (T1WI), fast spin-echo T2-weighted imaging (T2WI), and T2 fluid-attenuated inversion recovery (FLAIR) - to exclude brain parenchymal lesions, followed by functional 3D-pCASL acquisition using these parameters: TR 3722 ms, TE 94 ms, slice thickness 4 mm, FOV 24 × 24 cm, 36 slices, bandwidth 26.5 kHz, NEX 3, PLD 1525 ms, with a total scan time of 3 min 13 s. The 3D-pCASL image were conducted and analyzed by two board-certified radiologists (≥5 years’ neuroimaging experience). The abnormal areas of decreased or increased perfusion with bilateral comparisons of two contiguous slices were observed as positive foci. Any interpretive discrepancies were referred to a senior radiologist (associate professor-level or higher) for arbitration to ensure diagnostic consensus. Image interpretation was performed without knowledge of AEEG results.

### 2.5 Statistical analysis

All statistical analyses were conducted using Statistical Product and Service Solutions, version 26.0 (SPSS, Chicago, IL, USA). Qualitative data were presented as percentages (%) and verified using the chi-square test. Multivariate analysis was conducted using Generalized Estimating Equations (GEE). A *p*-value of <0.05 was considered indicative of a statistically significant difference.

## 3 Results

A total of 98 PWE were included in this study, with 49 males (50.00%) and 49 females (50.00%). The ages ranged from 10 to 58 years, the age of onset ranged from 1 to 56 years and the duration of illness ranged from 3 days to 33 years. The seizure types included 34 cases (34.69%) of focal seizures and 64 cases (65.30%) of generalized seizures.

### 3.1 The positive detection rates of different modalities

**Figure 1:**
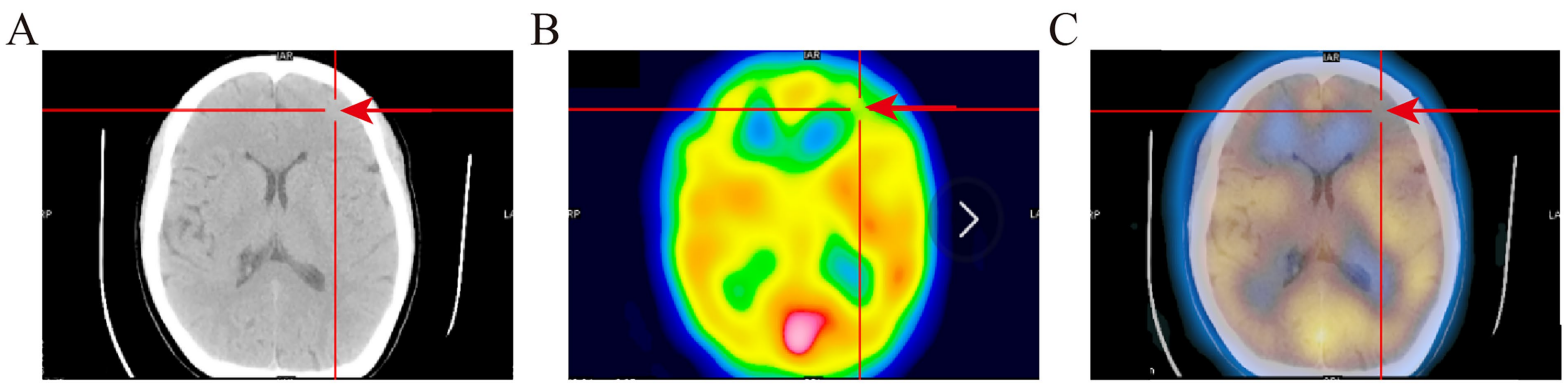
The SPECT/CT images of PWE. **(A)** The CT image. **(B)** The SPECT image. **(C)** The fusion image; Figure shows that the left frontal lobe has hypoperfusion than the contralateral side. Red arrow indicates ischemic lesion.

**Figure 2:**
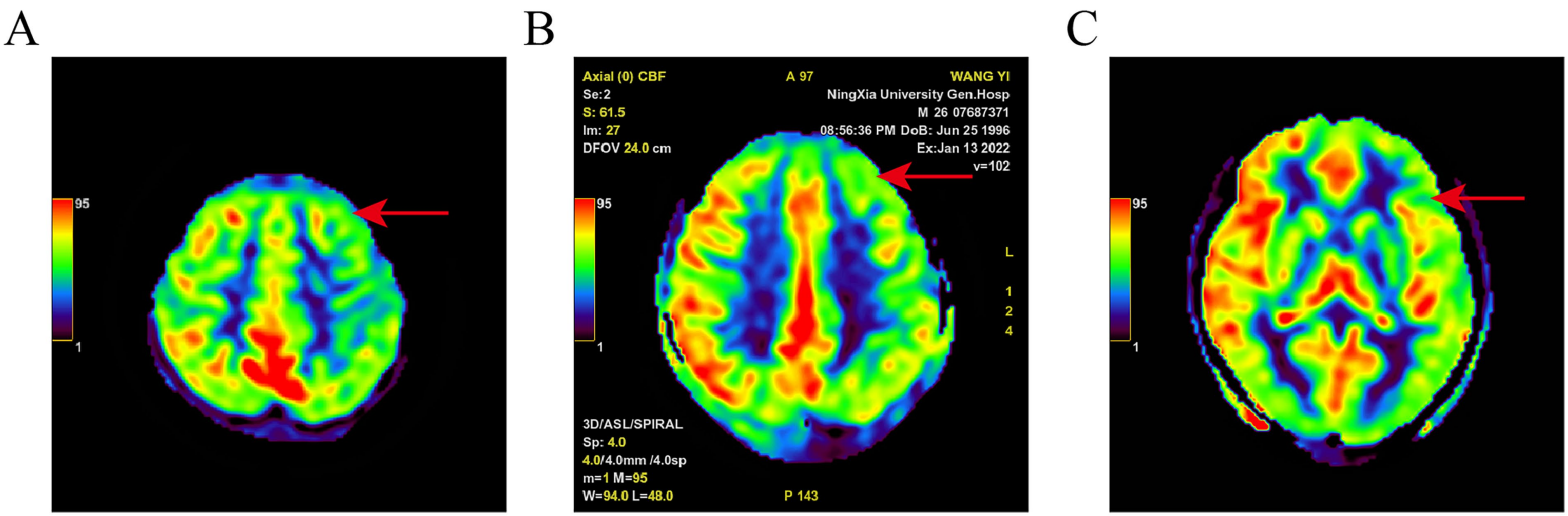
Pseudo-color map of ASL in a PWE. Different color bands and values in the upper left corner represent CBF from low to high; **(A)**: left frontal lobe hypoperfusion; **(B)**: left frontal lobe hypoperfusion; **(C)**: left temporal lobe hypoperfusion. Red arrow indicates ischemic lesion.

The positive result for SPECT/CT were reduced rCBF (Figure 1). The positive result for AEEG were the detection of IEDs. The positive result for 3D-pCASL were either reduced rCBF, increased rCBF, or coexisting increased and reduced rCBF (Figure 2). The positive detection rates for SPECT/CT, AEEG, and 3D-pCASL examinations were 77.55% (76/98), 62.24% (61/98), and 54.08% (53/98) respectively.

GEE analysis, after adjusting for sex, age at onset, duration of epilepsy, seizure type (focal/generalized), and seizure frequency, demonstrated: SPECT/CT exhibited higher positive detection rates than AEEG (aOR = 1.165, 95% CI: 1.036–1.311, *p* = 0.011) and 3D-pCASL (aOR = 1.265, 95% CI: 1.119–1.430, *p* < 0.001), no significant difference was observed between AEEG and 3D-pCASL (aOR = 0.922, 95% CI: 0.810–1.048, *p* = 0.213), sex, age at onset, duration of epilepsy, seizure type (focal/generalized), and seizure frequency showed no independent association with positive detection rates (Table 1).

**Table 1:** GEE analysis of modalities positive detection rates.

Variable	aOR	95%CI	*p-*Value	Comparison reference
Modality				
SPECT/CT vs. AEEG	1.165	1.036–1.311	0.011^*^	AEEG
3D-pCASL vs. AEEG	0.922	0.810–1.048	0.213	AEEG
SPECT/CT vs. 3D-pCASL	1.265	1.119–1.430	<0.001^*^	3D-pCASL
Clinical covariates				
Sex (Male vs. Female)	0.937	0.828–1.060	0.301	Female
Age at onset (per year)	0.998	0.993–1.003	0.510	
Disease duration (per year)	1.005	0.996–1.014	0.319	
Seizure type (Focal vs. Generalized)	1.014	0.887–1.160	0.837	Generalized
Seizure frequency grade				≤1 per year
>1 per year and ≤1 per month	1.085	0.946–1.244	0.244	
>1 per month	1.135	0.978–1.317	0.095	

aOR = adjusted odds ratio, CI = confidence interval. Asterisks (*) indicate statistically significant differences (*p* < 0.05).

In combined modalities, a result was defined as positive if any one of the three examinations (SPECT/CT, AEEG, or 3D-pCASL) indicated positivity. The positive detection rate of AEEG+SPECT/CT + 3D-pCASL (93.88%,92/98) was higher than AEEG+3D-pCASL (79.59%, 78/98, χ^2^ = 8.691, *p* = 0.003), but showed no statistically significant difference compared to AEEG+SPECT/CT (88.78%, 87/98, χ^2^ = 1.610, *p* = 0.204). No statistically significant difference in the positive detection rate was observed between the AEEG + SPECT/CT and the AEEG + 3D-pCASL (*χ*^2^ = 3.104, *p* = 0.078) (Table 2).

**Table 2:** The positive detection rates of combined modalities.

Comparison groups	Sample size (*N*)	Positive detection cases, *n* (%)	*p*-value
AEEG+SPECT/CT vs. AEEG+3D-pCASL	98	87(88.78) vs. 78(79.59)	0.078
AEEG+SPECT/CT vs. AEEG+SPECT/CT + 3D-pCASL	98	87(88.78) vs. 92(93.88)	0.204
AEEG+3D-pCASL vs. AEEG+SPECT/CT + 3D-pCASL	98	78(79.59) vs. 92(93.88)	0.003^*^

Asterisks (*) indicate statistically significant differences (*p* < 0.05).

### 3.2 Lesion characteristics in different modalities results

The results of SPECT/CT, AEEG, and 3D-pCASL showed different distribution of PWE with no lesions, focal lesions, unilateral multifocal lesions, and bilateral lesions (*χ*^2^ = 39.472, *p* < 0.001) (Table 3).

**Table 3:** Lesion characteristics in different modalities results.

Modality	No lesions *n* (%)	Focal lesions *n* (%)	Unilateral multifocal lesions *n* (%)	Bilateral lesions *n* (%)	*p*-value
SPECT/CT	22 (22.45)	19 (19.39)	32 (32.65)	25 (25.51)	<0.001^*^
AEEG	37 (37.76)	13 (13.27)	13 (13.27)	35 (35.71)	
3D-pCASL	45 (45.92)	25 (25.51)	22 (22.45)	6 (6.12)	

Asterisks (*) indicate statistically significant differences (*p* < 0.05).

### 3.3 The agreement between SPECT/CT, 3D-pCASL, and AEEG positive results

Among 61 PWE with IEDs detected by AEEG, the agreement rate between SPECT/CT and AEEG was 63.93% (39/61, including 4 cases of complete agreement and 35 cases of partial agreement), while that of 3D-pCASL was 39.34%(24/61, including 7 cases of complete agreement and 17 cases of partial agreement), the difference is statistically significant (*χ*^2^ = 7.385, *p =* 0.007) (Table 4).

**Table 4:** Agreement between SPECT/CT, 3D-pCASL, and AEEG positive results.

Modality	Complete agreement	Partial agreement	No agreement	Agreement rate, %(n1/n2)	*p-*value
SPECT/CT	4	35	22	63.93 (39/61)	0.007*
3D-pCASL	7	17	37	39.34 (24/61)	

Asterisks (*) indicate statistically significant differences (*p* < 0.05).

Among the 43 PWE with AEEG-identified IEDs in the temporal lobe, SPECT/CT detected concordant lesions in 31 PWE (72.09%, 31/43), significantly higher than 3D-pCASL in 11 PWE (25.58%, 11/43, *χ*^2^ = 18.651, *p* < 0.001). Among the 31 PWE with AEEG-identified IEDs in the frontal lobe, SPECT/CT detected concordant lesions in 17 PWE (54.84%, 17/31), significantly higher than 3D-pCASL in 8 PWE (25.81%, 8/31, *χ*^2^ = 5.429, *p* = 0.020). Among the 12 PWE with AEEG-identified IEDs in the generalized widespread, SPECT/CT detected concordant lesions in 4 PWE (33.33%, 4/12), compared to 2 PWE (16.67%, 2/12) for 3D-pCASL, with no statistically significant difference (*p* = 0.640) (Table 5).

**Table 5:** Agreement between 3D pCASL, SPECT/CT and location of IEDs.

Location of IEDs	SPECT/CT n/%	3D-pCASL n/%	*p-*value
Temporal lobe (*N* = 43)	31 (72.09)	11 (25.58)	<0.001^*^
Frontal lobe (*N* = 31)	17 (54.84)	8 (25.81)	0.020^*^
Generalized widespread (*N* = 12)	4 (33.33)	2 (16.67)	0.640
Other (*N* = 13)	4 (30.78)	0	—

“—” means that no statistical test was performed because of the small sample size. Asterisks (*) indicate statistically significant differences (*p* < 0.05).

## 4 Discussion

Distinct patterns of rCBF alterations manifest in brain tissues during both ictal and interictal periods and monitoring these rCBF variations is relevant for the diagnosis, treatment and management of epilepsy (11).

The interictal SPECT/CT demonstrated a positive detection rate of 77.55% (76/98), with all positive findings manifesting rCBF reduction in our study. This finding aligns with prior studies (5, 12). Recurrent seizures may lead to cerebral hypoxia, ischemia and excessive oxygen free radicals, damaging neurons, thereby resulting in reduced perfusion in lesion as reflected by decreased rCBF. The interictal 3D-pCASL demonstrated a positive detection rate of 54.08% (53/98). Positive findings manifested as three distinct hemodynamic patterns: rCBF decrease, rCBF increase, or coexisting rCBF increases and decreases. The observed patterns of rCBF increase and coexisting rCBF increases and decreases appear incongruent with classical interictal hypoperfusion theory. Studies comparing perfusion in epileptic focus and contralateral brain regions in temporal lobe epilepsy revealed “inverse perfusion changes” during interictal and postictal phases: increased rCBF stimulation induced hypoperfusion in epileptic focus but hyperperfusion in contralateral regions. Conversely, extra-temporal epilepsy manifests perfusion increases in epileptic focus during rCBF augmentation, with relative contralateral reduction (13, 14), potentially explaining the increased rCBF and mixed rCBF patterns observed in 3D-pCASL.

AEEG is commonly used to classify epilepsy syndromes by capturing IEDs (15). Significantly higher detection rates were observed with SPECT/CT compared to both AEEG (62.24%, 61/98) and 3D-pCASL. No significant difference emerged between AEEG and 3D-pCASL detection rates. IEDs constitute an established electrophysiological biomarker of epileptogenic foci, generated by synchronous neuronal population activity. IEDs represent heterogeneous pathological phenomena closely associated with seizure genesis (16). Alterations in rCBF closely track electrophysiological effects of ictal epileptiform discharges or IEDs (17), indicating that SPECT/CT- and 3D-pCASL-measured rCBF dynamics are intrinsically coupled with IEDs-related neurophysiological events. Notably, 3D-pCASL-derived hyperperfusion and mixed perfusion patterns warrant particular attention, as evidenced by studies demonstrating concordance between ASL hyperperfusion zones and validated epileptogenic foci (18). AEEG localizes electrophysiological abnormalities, while perfusion imaging delineates the extent of metabolic alterations. Cases demonstrating AEEG-negative but perfusion-positive findings may indicate metabolically active epileptogenic zones without surface-detectable IEDs. Discordant perfusion findings (e.g., SPECT hypoperfusion with ASL hyperperfusion) likely reflect distinct metabolic states across temporal phases, particularly when ASL acquisition occurs temporally closer to ictal onset. The lower positive rate of 3D-pCASL compared to SPECT/CT in this study deserves our attention. 3D-pCASL employs a series of short, high-frequency radiofrequency pulses to invert arterial blood magnetization in a pseudo-steady-state or adiabatic manner (19). This technique demonstrates high reproducibility and repeatability, establishing it as a reliable method for clinical image acquisition (20). In this study, the lowest positive detection rate of epileptic foci in 3D pCASL examination of PWE may be due to artifact interference and low labeling efficiency.

The positive detection rates of SPECT/CT, AEEG, and 3D-pCASL examinations differed among PWE in our study. Each of three modalities has its own advantages and limitations in clinical application. Although SPECT/CT demonstrated the highest positive detection rate, its associated radiation exposure cannot be overlooked—particularly since the combined acquisition of functional and anatomical images in SPECT/CT increases radiation burden to patients. This poses limitations to its application (21). AEEG is characterized by its low cost, convenience, and relatively high diagnostic yield. Additionally, it is not only capable of detecting IEDs but also enables the quantification of seizure, facilitating personalized treatment strategies (22, 23). 3D-pCASL, as a non-invasive technique, holds potential for widespread use due to its ability to perform repeated measurements during the interictal, ictal phases. Nonetheless, its cost is higher compared to AEEG and SPECT/CT. In the combined modalities, the positive detection rate of AEEG+SPECT/CT + 3D-pCASL in PWE reached 93.88% (92/98), which was significantly higher than AEEG+3D-pCASL in this study and also exceeded the rates reported in previous studies for both individual or multiple modalities Combination. While the advantage of multiple modalities combination is undeniable, it also significantly increases the financial burden on PWE, which may lead to challenges for clinical implementation. However, the positive detection rate serves merely as a foundational metric for evaluating diagnostic utility; the precision of lesion localization constitutes the core determinant of clinical value (e.g., in presurgical evaluation). The absence of surgical validation in our cohort limits definitive assessment of localization accuracy. Future studies correlating multimodal imaging with stereo-EEG and resection outcomes will be essential to establish validated localization criteria.

The analysis of agreement between SPECT/CT, 3D-pCASL, and AEEG-identified IEDs (61 PWE) revealed higher SPECT/CT-AEEG agreement (63.93%, 39/61) compared to 3D-pCASL (39.34%, 24/61). Consistent neuroimaging localization across modalities strengthens epileptic focus identification, supporting SPECT/CT’s superior accuracy, as noted previously (24). Among 43 PWE with AEEG-identified temporal lobe discharges, SPECT/CT detected agreement areas in 31 cases (72.09%, 31/43), significantly outperforming 3D-pCASL (25.58%, 11/43). The complex anatomy of the medial temporal lobe, surrounded by cerebrospinal fluid and vasculature, may impair 3D-pCASL’s rCBF monitoring and localization accuracy.

Overall, this study provides a comparative analysis of SPECT/CT, AEEG and 3D-pCASL in PWE, and provided some factors that can be considered in clinical application. The integration of multimodal neuroimaging has become increasingly pivotal in presurgical evaluation for drug-resistant epilepsy. While encompassing diverse techniques, its implementation requires context-specific integration of multiple diagnostic findings to enable comprehensive clinical inference. This approach enhances the utility of preoperative assessments and improves localization accuracy of epileptogenic zones, thereby increasing surgical candidacy rates and optimizing postoperative outcomes for drug-resistant epilepsy patients (25). Although the lack of surgical validation in our cohort limits definitive assessment of localization accuracy, our findings may still serve as a suggestive indicator for preoperative localization in PWE. When a patient with drug-resistant epilepsy presents with a negative MRI, the triad of clinical presentation, medical history, and highly agreement AEEG, SPECT/CT, and 3D-pCASL findings may suggest a resectable epileptogenic zone. In our study, findings in newly diagnosed PWE are also noteworthy. Among the 6 newly diagnosed PWE, 4 had negative AEEG results. In these 4 PWE, SPECT/CT showed positive findings in all cases, while 3D-pCASL was positive in one case. As one diagnostic criterion for epilepsy requires one unprovoked (or reflex) seizure and a probability of further seizures similar to the general recurrence risk (at least 60%) after two unprovoked seizures, occurring over the next 10 years (26). These observations suggest that positive SPECT/CT or 3D-pCASL findings may indicate an elevated recurrence risk and potentially contribute to epilepsy diagnosis. However, due to the limited sample size, formal risk assessment was precluded. Future studies should investigate SPECT/CT and 3D-pCASL in patients with suspected epilepsy to validate these preliminary findings. Current withdrawal criteria for anti-seizure medications (ASMs) in PWE are relatively standardized (27). However, studies report an overall seizure recurrence rate of 23.75% after drug withdrawal (28). When PWE meet reasonable seizure-free period with negative EEG monitoring but demonstrate perfusion abnormalities on SPECT/CT or 3D-pCASL, deferring ASMs withdrawal may mitigate recurrence risk. Future research should focus on pre-withdrawal SPECT/CT and 3D pCASL assessments to further investigate this potential association. In the future, we believe that SPECT/CT and 3D-pCASL will be applied more effectively in PWE, providing a solid theoretical basis for diagnosis, treatment, and prognostic evaluation.

Several study limitations warrant acknowledgment. First, the absence of surgical or pathological validation as a gold standard limits our ability to confirm the localization accuracy of positively identified lesions. Second, the single-center design further constrains result reliability and generalizability. Third, potential selection bias may have been introduced during patient enrollment. Moreover, the visual analysis methods employed in 3D-pCASL and SPECT/CT for assessing rCBF perfusion and localized functional changes are relatively crude. These approaches heavily rely on the clinical experience of nuclear medicine specialists, radiologists, and researchers. In summary, a analysis of SPECT/CT, AEEG, and 3D-pCASL were conducted in PWE. The results demonstrated that SPECT/CT exhibited the highest positive detection rate and showed superior agreement with AEEG than 3D-pCASL. Furthermore, the combined use of SPECT/CT, AEEG and 3D-pCASL significantly enhanced the positive detection rate, suggesting the advantages of SPECT/CT and combined modalities.

## Data Availability

The raw data supporting the conclusions of this article will be made available by the authors, without undue reservation.
